# The Temporal Dynamics of Coastal Phytoplankton and Bacterioplankton in the Eastern Mediterranean Sea

**DOI:** 10.1371/journal.pone.0140690

**Published:** 2015-10-16

**Authors:** Ofrat Raveh, Niv David, Gil Rilov, Eyal Rahav

**Affiliations:** National Institute of Oceanography, Israel Oceanographic and Limnological Research, Haifa, Israel; University of Connecticut, UNITED STATES

## Abstract

This study considers variability in phytoplankton and heterotrophic bacterial abundances and production rates, in one of the most oligotrophic marine regions in the world–the Levantine Basin. The temporal dynamics of these planktonic groups were studied in the coastal waters of the southeastern Mediterranean Sea approximately every two weeks for a total of two years. Heterotrophic bacteria were abundant mostly during late summer and midwinter, and were positively correlated with bacterial production and with N_2_ fixation. Based on size fractionating, picophytoplankton was abundant during the summer, whereas nano-microphytoplankton predominated during the winter and early spring, which were also evident in the size-fractionated primary production rates. Autotrophic abundance and production correlated negatively with temperature, but did not correlate with inorganic nutrients. Furthermore, a comparison of our results with results from the open Levantine Basin demonstrates that autotrophic and heterotrophic production, as well as N_2_ fixation rates, are considerably higher in the coastal habitat than in the open sea, while nutrient levels or cell abundance are not different. These findings have important ecological implications for food web dynamics and for biological carbon sequestration in this understudied region.

## Introduction

The Levantine Basin of the eastern Mediterranean Sea is one of the most oligotrophic marine environments in the world [1, 2, 3]. Dissolved inorganic nitrogen and phosphorus are usually observed at low [4] concentrations, suggesting severe nutrient limitation of autotrophic and possibly heterotrophic bacterial growth rates [5]. Concurrently, low chlorophyll *a* (Chl a) concentrations (<0.2 μg L^-1^) [6, 7, 8], low primary production (~200 mg C m^-2^ d^-1^) [9, 10], and low N_2_ fixation (<0.2 nmol N L^-1^ d^-1^) [8] rates are usually found at the surface. The phytoplankton biomass is often dominated by small pico-autotrophic cells (<3 μm) [5] due to their large surface area to volume ratios, which may allow them to utilize the low concentrations of ambient nutrients faster than larger microphytoplankton such as diatoms and dinoflagellates (>20 μm) [11, 12]. These pico-sized organisms are responsible for approximately 60% of the Chl a and 65% of the primary annual production in the pelagic eastern Mediterranean Sea areas [13], making up as much as 80% of the biomass off the Israeli coastline during the early 1980s [14].

Heterotrophic bacteria also play an important role in the upper layers of the open oligotrophic Mediterranean Sea [15, 16], with bacterial abundance and production reaching ~10^5^ cells mL^-1^ and <1 μg C L^-1^ d^-1^ respectively [5, 15]. Nevertheless, these organisms are responsible for the majority of the nutrient recycling in this low nutrient, low chlorophyll system [15, 17], as is typical for other oligotrophic environments [18, 19].

The coastal waters of the eastern Mediterranean Sea are considered to be more productive environments than the open sea, mainly due to occasional water runoff from land, wind, precipitation and anthropogenic pressure. These inputs can greatly influence microbial production and diversity. Yet, our understanding of the seasonal changes in the abundance and production of phytoplankton and bacteria in the easternmost oligotrophic coast of the Levantine Basin is incomplete, and most of the existing research was conducted 20 to 30 years ago, at stations located a few kilometers off the Israeli coast [1, 14, 20]. Since then, the surface temperature in the Levantine Basin (eastern Mediterranean Sea) rose by ~3°C [21], which may alter microbial populations (whether positively or negatively) and thus affect the bottom of the food web.

In this study, we have measured the surface temporal distribution and production of phytoplankton and heterotrophic bacteria and of N_2_ fixation rates in the coastal ultraoligotrophic waters of the Levantine Basin for two consecutive years (April 2013 to April 2015). Our objectives were: (*i*) to describe the seasonal development of autotrophic and heterotrophic bacterial abundance at a Levantine Basin coastal station, (*ii*) to quantify the *in-situ* daily primary production and bacterial production and determine the relative importance of cell size to this process, (*iii*) to determine the role of diazotrophy (dinitrogen fixation) in this nutrient-poor environment, and (*iv*) to identify the factor(s) that affects the abundance and the production of these microorganisms in the Levantine Basin.

## Materials and Methods

### Study site and sampling

This study was conducted between April 2013 and April 2015 in the coastal waters of Tel Shikmona (Haifa),approximately 50 m from the Israel Oceanographic and Limnological Research (IOLR) Institute (32°49′34N, 34°57′20E) (bottom depth ~5 m). Surface water (2 m) samples were collected by pumping, approximately every two weeks, except for in the case of inorganic nutrients, which were measured monthly. The pumped seawater was distributed into three acid-cleaned Nalgene bottles (4.6 L each) and was immediately brought to the lab for further subsampling for the different analyses described below. The temperature was measured using an *in situ* HOBO Pendant Temperature data logger (model UA-002-64, Onset Computer Corporation) mounted on the rocky bottom at the same depth. Salinity was measured using a Yellow Spring Instruments YSI 6000. Note that no specific permissions were required for the operation of this experiment and thus this study did not involve endangered or protected species

### Inorganic nutrients

Water samples were collected monthly in 15–mL acid-washed plastic scintillation vials and placed immediately in a -20°C freezer until analysis. Nutrient content was determined from 1–2 plastic scintillation vials using the segmented flow Seal Analytical AA-3 system described by Krom et al. [22] and Kress & Herut [23] within 3–6 months of collection. The limits of detection (twice the standard deviation of the blank) were 0.08 μM for nitrate+ nitrite, 0.008 μM for phosphate, and 0.05 μM for silicic acid.

### Chlorophyll *a* (Chl a)

Seawater was collected into two 300–500 mL bottles and passed through 0.7-μm glass fiber filters in order to isolate all phytoplankton Chl a, and 3-μm glass fiber filters (Pall Inc) were used to separate the nano and microphytoplankton (hereafter referred to collectively as nano-microphytoplankton) contribution of Chl a from the smaller-size phytoplankton. Filters were stored at -20°C, in a light-tight box. Samples were extracted overnight in 5 mL of 90% acetone at 4°C in the dark within 1 week after collection. Chl a concentrations were determined using a Luminescence Trilogy Spectrofluorometer with a 436-nm excitation filter and a 680-nm emission filter [24]. The differences in fluorescence between the two filter types (0.7 and 3 μm) (i.e., total phytoplankton minus the contribution of nano-microphytoplankton) were defined as the picophytoplankton Chl a contribution. The phytoplankton size distribution upon which the described analyses were performed is based on Chisholm [25]. We are aware that the use of glass fiber filters may not be as quantitative as polycarbonate membranes may be for specific cell sizes. However, due to the extraction method, glass fiber filters were chosen.

### Pico-phytoplankton and bacterial abundance

Duplicate pico-phytoplankton samples (1.8 mL), taken from the same bottles the Chl a samples were drawn from, were fixed with 50% glutaraldehyde (6 μl, Sigma-Aldrich G7651) and stored at -80°C until analysis, usually performed within 1 week or less. Pico-phytoplankton abundance was determined using an Attune® Acoustic Focusing Flow Cytometer (Applied Biosystems) equipped with a syringe-based fluidic system and 488 and 405-nm lasers. Taxonomic discrimination was based on the orange fluorescence of phycoerythrin (585 nm) and the red fluorescence of Chl a (630 nm) [26], on side-scatter (SSC, a proxy of cell volume) [27], and on forward-scatter (FSC, a proxy of cell size) [28]. A 1-μm bead (Polysciences) was used as a standard [29]. Samples were fast-thawed at 37°C in a water bath, and the abundance of pico-phytoplankton (i.e. *Synechococcus*, *Prochlorococcus* and small eukaryotes) was determined. *Prochlorococcus* abundance was usually very low throughout the study period, whereas *Synechococcus* comprised the majority of the picophytoplankton in most of our samples (S1 Table). Thus, Synechoco*ccus* and *Prochlorococcus* were merged and are henceforth referred to as “cyanobacteria”.

For heterotrophic bacterial abundance, 300 μL of the fixed water samples used for the picophytoplankton and nano-microphytoplankton determination were separately incubated at room temperature with the nucleic acid stain SYTO9 (1:10^5^ vol:vol) for 10 min in the dark [26] and then run at a low flow rate of 25 μL min^-1^ using a discrimination threshold of green fluorescence (520 nm).

### Primary production (PP)

The photosynthetic carbon fixation rates were estimated using the ^14^C incorporation method [30]. Water samples were analyzed in triplicates with dark and zero-time controls. Samples (50 mL) collected at 09:00 AM were added to polycarbonate bottles (Nalgene) containing 5 μCi of NaH^14^CO_3_ (Perkin Elmer) and incubated for 4–5 h under ambient natural illumination and temperature. To determine the quantity of the added radioactivity, 50 μL of each sample were immediately mixed with 50 μL of ethanolamine and stored for analysis. The incubations were terminated by filtering the spiked seawater onto GF/F filters in order to separate the total primary production or onto a 3-μm glass fiber filter in order to determine the contribution of nano-microphytoplankton to primary production at <50 mmHg. The filters were incubated overnight in 5 mL scintillation vials containing 50 μl of 32% HCl in order to remove excess ^14^C-bicarbonate. After adding 5 mL of scintillation cocktail (Ultima-Gold) to each vial, the radioactivity was measured using a TRI-CARB 2100 TR (Packard) liquid scintillation counter.

### Bacterial production (BP)

Bacterial production was estimated using the [4,5-^3^H]-leucine incorporation method (Amersham; specific activity: 160 Ci mmoL^-1^) [31]. Three aliquots (1.7 mL each) from each water sample (i.e. a total of nine tubes for each measurement) were incubated with 100 nmol leucine L^-1^ for 4–5 h at an ambient temperature in the dark. Preliminary experiments indicated that this was a saturating level of ^3^H-leucine and that incorporation was linear during this time (data not shown). A triplicate addition of trichloroacetic acid (TCA) served as a control. The incubations were terminated with 100 μL of cold (4°C) TCA (100%), followed by the micro-centrifugation protocol [32]. After adding 1 mL of scintillation cocktail (Ultima-Gold) to each vial, the samples were counted using a TRI-CARB 2100 TR (Packard) liquid scintillation counter. We used a conversion factor of 3 kg C mol^-1^ per mole leucine incorporated, assuming an isotopic dilution of 2.0 [33].

### Dinitrogen (N_2_) fixation

Dinitrogen fixation rates were measured in triplicates using the newly developed ^15^N_2_-enriched seawater protocol [34]. ^15^N_2_ enriched seawater was prepared by injecting 1:100 (vol:vol) ^15^N_2_ gas (99%) into degassed (MiniModule G543) and filtered (Sterivex 0.2 μm) seawater collected at the study site. The enriched seawater stock was shaken vigorously in order to completely dissolve the ^15^N_2_ gas and aliquots (225 mL) were then added to the triplicate experimental bottles (4.6 L). Following 24 h of incubations under ambient light and temperature, the samples were filtered through pre-combusted (450°C, 4.5 h) 25 mm GF/F filters and dried in an oven at 60°C overnight. The samples were then analyzed using a CE Instruments NC2500 elemental analyzer interfaced to a Thermo-Finningan Delta Plus XP isotope ratio mass spectrometer (IRMS). For isotope ratio mass spectrometry, a standard curve to determine N mass was performed with each sample run.

The percentage of N_2_ fixation contributed to the primary production was calculated based on the averaged particulate organic carbon (POC) and nitrogen (PON) concentration found in the eastern Mediterranean Sea, as described in the details in Yogev et al. [35]. Based on previous studies in this system, the average measured POC:PON ratio was used (i.e. 8) instead of the “Redfield” ~6.6 ratio, as it better characterizes this system [35].

### Statistical analysis

Data is displayed as an average, with error bars signifying one standard deviation (n = 2 to 3). The differences between coastal and compiled open sea data were evaluated using a one-way analysis of variance (ANOVA), followed by a Fisher LSD multiple comparison post hoc test with a confidence of 95% (α- 0.05), performed using the XLSTAT software.

## Results

### The temporal variability of autotrophic and heterotrophic bacteria

**Fig 1:**
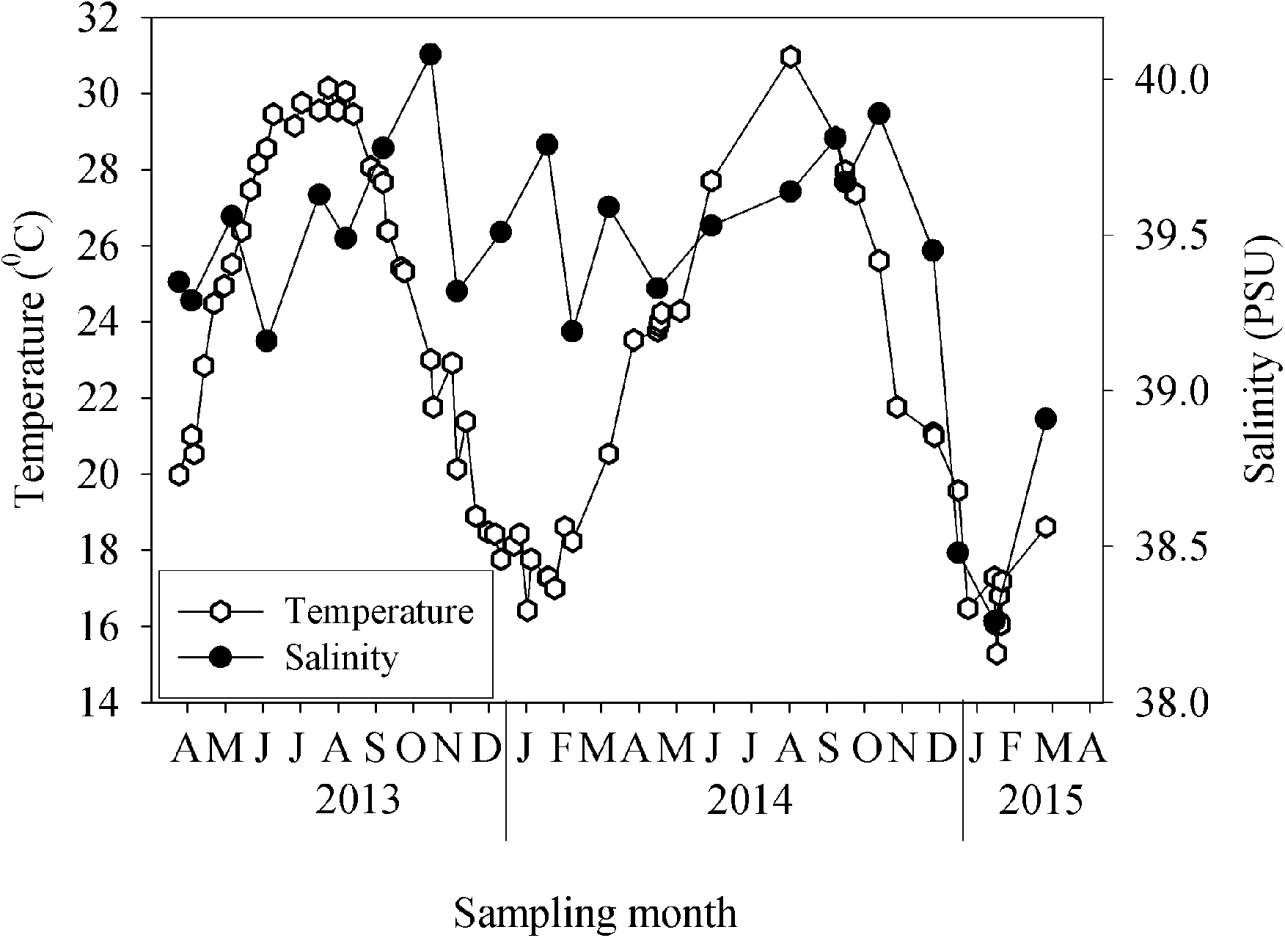
The seasonal surface (2 m) variability of temperature (open circle) and salinity (black circle). Data were collected between April 2013 and April 2015 at the study site off the eastern Mediterranean coast.

The seasonal characteristics of temperature and of inorganic nutrients in the nearshore waters of Tel Shikmona between April 2013 and April 2015 are shown in Fig 1 and Table 1. Temperatures were as low as ~16°C in wintertime (January and February of 2014 and 2015) and increased to >30°C during the summer months (August and September of 2013 and 2014). The level of salinity did not exhibit any distinct seasonal variability and was usually > 39.0 PSU, except during the winter of 2015 when lower salinity levels were recorded (Fig 1). The concentrations of inorganic nutrients (e.g., nitrate+ nitrite, phosphorus, and silicic acid) throughout the study period were in the nano-molar range and did not reveal any distinct seasonal trends (Table 1). Dissolved NO_2_+NO_3_ were low (0.20± 0.11 μM) overall, especially during the early summer and autumn of 2014 (<0.10 μM), though they were somewhat higher during January and February of 2015 (> 1 μM). Phosphorus concentrations were low throughout the sampling period (0.02± 0.01 μM), with the highest concentrations measured during both winters (~0.08 μM) and the lowest measured at the end of both summers (below the detection limit). Exceptionally high phosphate concentrations were recorded following a sewage effluent discharge during February 2015 (Table 1). The concentration of silicic acid (Si(OH)_4_) was the lowest during midwinter (February 2014, 0.30 μM) and the highest during June 2014 (2.39 μM).

**Table 1:** The chemical characteristics of surface seawater (~2 m deep) sampled between April 2013 and April 2015. The limits of detection (twice the standard deviation of the blank) were 0.08 µM for nitrate+ nitrite, 0.008 μM for phosphate, and 0.05 μM for silicic acid. BDL: below detection limit, NA: not available.

Sampling period	NO_2_+NO_3_ (μM)	PO_4_ (μM)	Si(OH)_4_ (μM)
*2013*			
Apr.	0.29	0.04	1.72
May	0.28	0.04	1.01
Jun.	0.20	0.02	1.26
Jul.	0.21	0.03	1.54
Aug.	0.26	BDL	1.16
Sept.	0.37	0.01	1.32
Oct.	BDL	BDL	0.64
Nov.	BDL	0.02	0.84
Dec.	0.30	0.01	2.12
*2014*			
Jan.	0.26	0.03	0.64
Feb.	0.24	0.02	0.30
Mar.	0.19	0.04	0.59
Apr.	NA	0.04	0.76
May	0.12	0.02	0.67
Jun.	0.10	0.01	2.39
Jul.	0.26	0.01	1.71
Aug.	0.36	0.02	1.30
Sept.	0.23	0.03	1.81
Oct.	0.16	BDL	0.94
Nov.	0.20	0.02	1.56
Dec.	0.24	BDL	1.00
*2015*			
Jan.	1.21	0.05	1.46
Feb.^*^	1.37–2.49	0.08–0.29	1.12–2.18
Mar.	0.64	0.01	0.86
Apr.	0.14	0.01	0.90

*A range provided using both the routine sampling and the measurements taken during the sewage effluent discharge.

**Fig 2:**
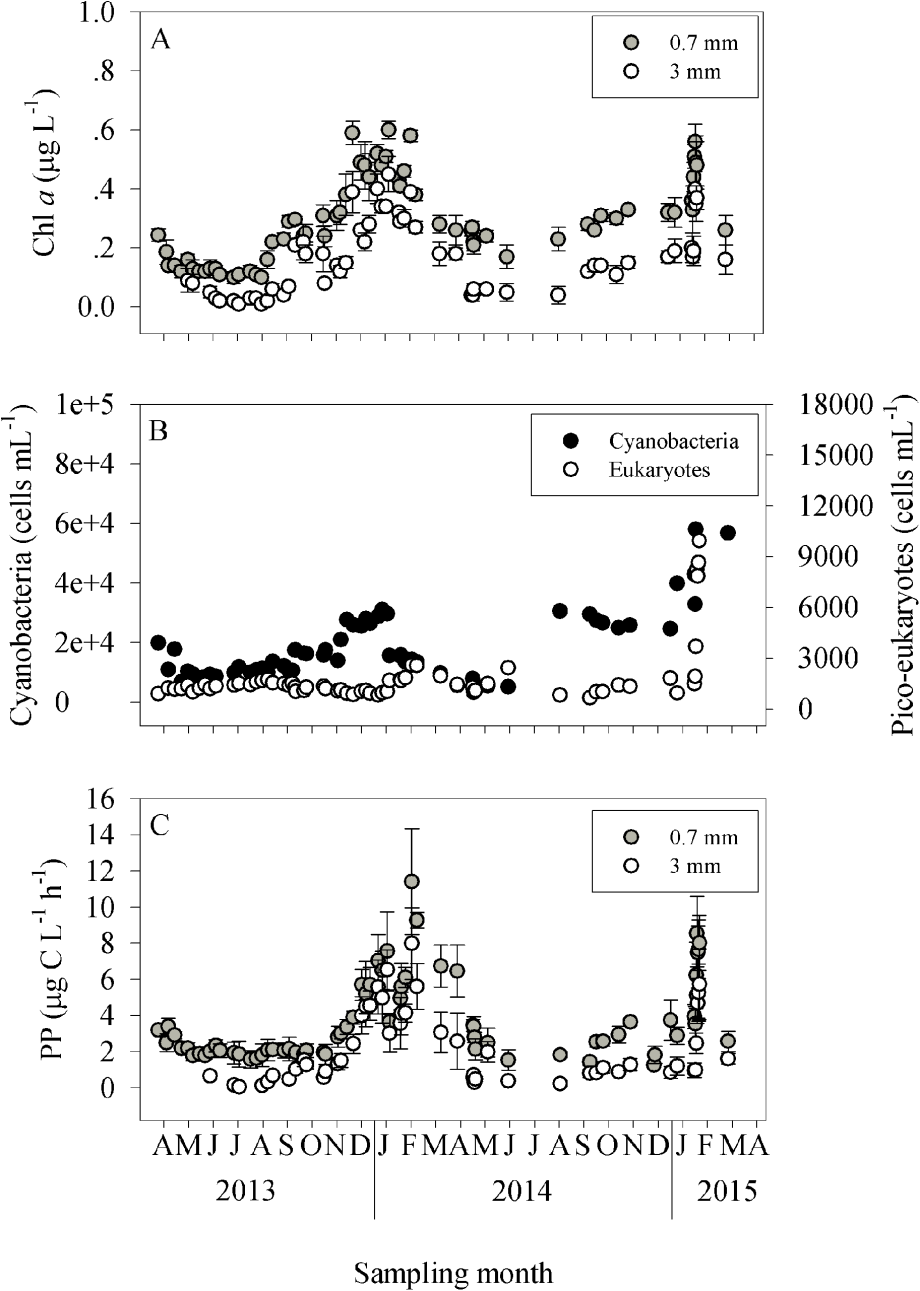
The temporal dynamics of autotrophic bacterioplankton in the coastal eastern Mediterranean Sea. Data presented are for Chl a (A) pico-phytoplankton (B) and primary production, PP (C) between April 2013 and April 2015.

The total chlorophyll *a* (Chl a) levels (filtered onto 0.7 μm GF/F) showed clear seasonal dynamics, with the lowest concentrations measured during summer (~0.10 μg L^-1^) and the peak concentrations measured during wintertime (~0.55 μg L^-1^) (Fig 2A). The nano-microphytoplankton Chl a (filtered onto a 3 μm filter) concentrations displayed similar seasonal patterns, ranging from 0.01 μg L^-1^ in July and August of 2013 to 0.45 μg L^-1^ in January 2014 (Fig 2A). The relative contribution of nano-microphytoplankton Chl a was high during both winters (~60%) and significantly lower during both summers, usually below 30% (Table 2). The abundance of cyanobacteria (i.e., *Synechococcus* and *Prochlorococcus*) and picoeukaryotes derived from flow-cytometric analyses was overall throughout both 2014 and 2015 (Fig 2B, S1 Table), and followed the same pattern as Chl a (Fig 2A). Concurrent with the abundances, the contribution of picophytoplankton Chl a was highest during the summer (57–91%) and the lowest during winter (<50%, Table 2).

**Table 2:** The average and range (in parenthesis) of chlorophyll *a* (Chl a) and the primary production (PP) distribution for each size fraction as a percentage of the total values obtained from Tel Shikmona from between April 2013 and April 2015. NA: not available. The raw data can be found in the supplementary (S1 Table).

Sampling period	Chl a (% of the total)		PP (% of the total)	
	Picophytoplankton (0.7–3 μm)	Nano-microphytoplankton (> 3 μm)	Picophytoplankton (0.7–3 μm)	Nano-microphytoplankton (> 3 μm)
*2013*				
Apr.	NA	NA	NA	NA
May	44	56	NA	NA
Jun.	50± 16 (38–62)	50± 16 (38–62)	68	32
Jul.	82± 6 (77–91)	18± 6 (9–23)	95± 4 (92–97)	5± 4 (3–8)
Aug.	79± 9 (73–90)	21± 9 (10–27)	92	8
Sept.	80± 7 (73–88)	20± 7 (12–27)	76± 8 (67–84)	24± 8 (16–33)
Oct.	22± 12 (28–29)	78 ± 12 (71–72)	34± 15 (17–47)	66± 15 (53–83)
Nov.	54± 12 (42–67)	46± 12 (33–58)	58± 11 (51–70)	42± 11 (30–49)
Dec.	52± 12 (34–63)	48± 12 (37–66)	33± 15 (13–37)	67± 15 (63–87)
*2014*				
Jan.	29± 6 (23–36)	71± 6 (64–77)	19± 4 (14–21)	81± 4 (79–86)
Feb.	31± 4 (26–35)	69± 4 (65–74)	29± 3 (27–32)	71± 3 (68–73)
Mar.	29	71	39	61
Apr.	33± 3 (31–36)	67± 3 (64–69)	57± 4 (58–64)	43± 4 (36–42)
May	80± 6 (71–85)	20± 6 (15–29)	70± 28 (78–89)	30± 28 (11–22)
Jun.	75	25	75	25
Jul.	71	29	NA	NA
Aug.	83	17	87	13
Sept.	57	43	44	56
Oct.	50± 6 (46–55)	50± 6 (45–54)	62± 7 (57–67)	38± 7 (33–43)
Nov.	59± 6 (55–63)	59± 6 (37–45)	67± 4 (65–70)	33± 4 (30–35)
Dec.	NA	NA	NA	NA
*2015*				
Jan.	45± 3 (41–47)	55± 3 (53–59)	68± 12 (33–42)	32± 12 (58–77)
Feb.	38± 13 (23–57)	62± 13 (43–77)	50± 19 (29–75)	50± 19 (25–71)
Mar.	38	62	36	64
Apr.	39± 2 (37–41)	61± 2 (59–63)	39	61

Similar to Chl a, photosynthetic carbon fixation rates (i.e. primary production) also exhibited seasonal patterns throughout the sampling period, with the highest rates measured in winter and the lowest in midsummer (Fig 2C). The relative contribution of nano-microphytoplankton to the total primary production was largely dependent on the period of sampling (Table 2). In general, nano-microphytoplankton made a greater contribution to the total primary production levels during winter (up to 87%), while picophytoplankton primary production became the primary contributor under the ultraoligotrophic summer conditions (up to 97%).

**Fig 3:**
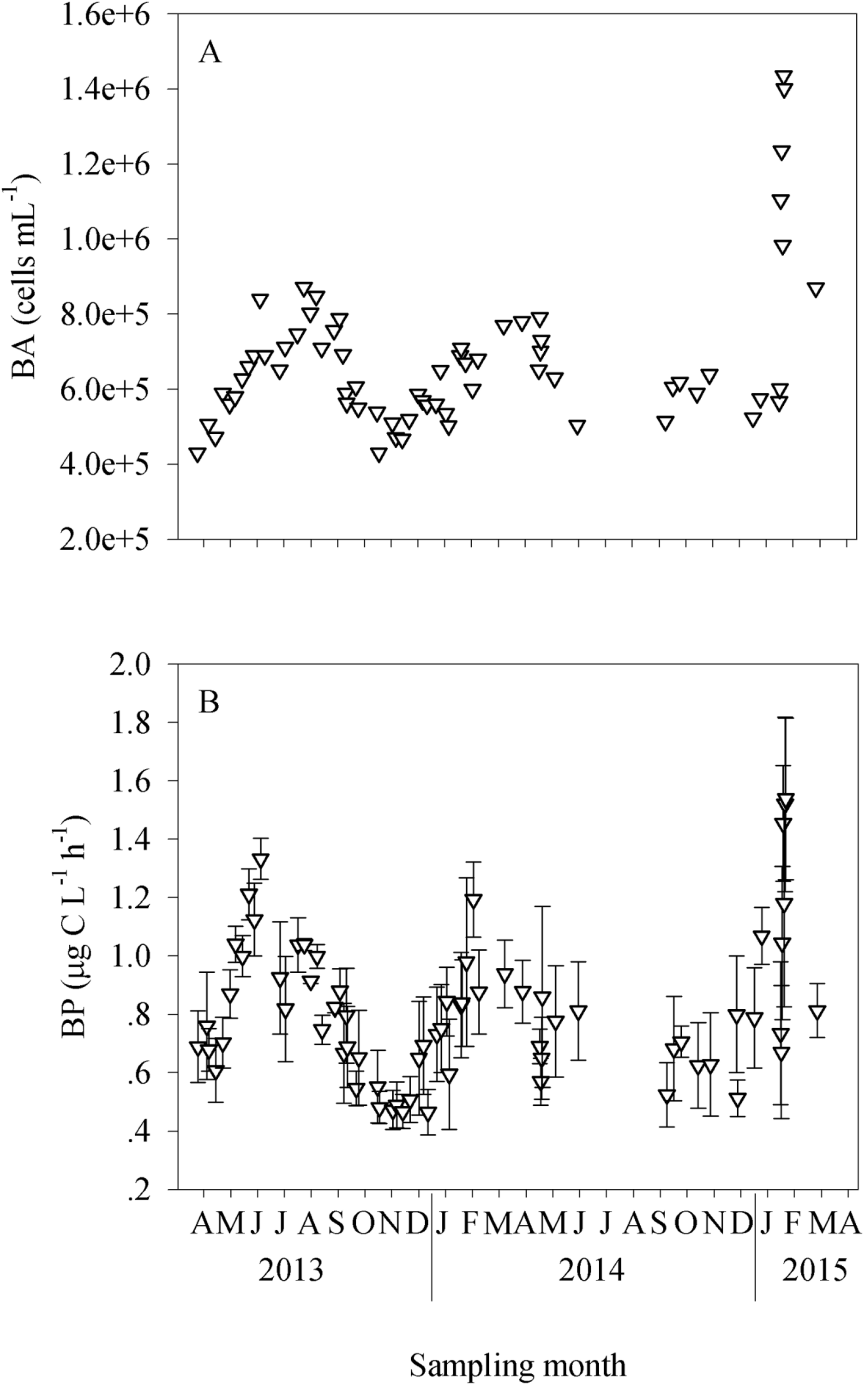
The temporal dynamics of heterotrophic bacterioplankton in the coastal eastern Mediterranean Sea. Data presented are for bacterial abundance, BA (A) and bacterial production, BP (B) between April 2013 and April 2015.

The abundance of heterotrophic bacteria ranged between 4.3 × 10^5^ and 1.4 × 10^6^ cells mL^-1^, with the highest abundances measured during February 2015, when an anthropogenic sewage input was recorded, and the lowest during the autumn (November 2013) (Fig 3A). Bacterial production followed the same trend as the bacterial abundance, with the highest rates measured during midsummer and midwinter (~1 μg C L^-1^ d^-1^), before they decreased by a factor of 2–4 during the spring and autumn of both years (~0.5 μg C L^-1^ d^-1^, Fig 3B).

**Fig 4:**
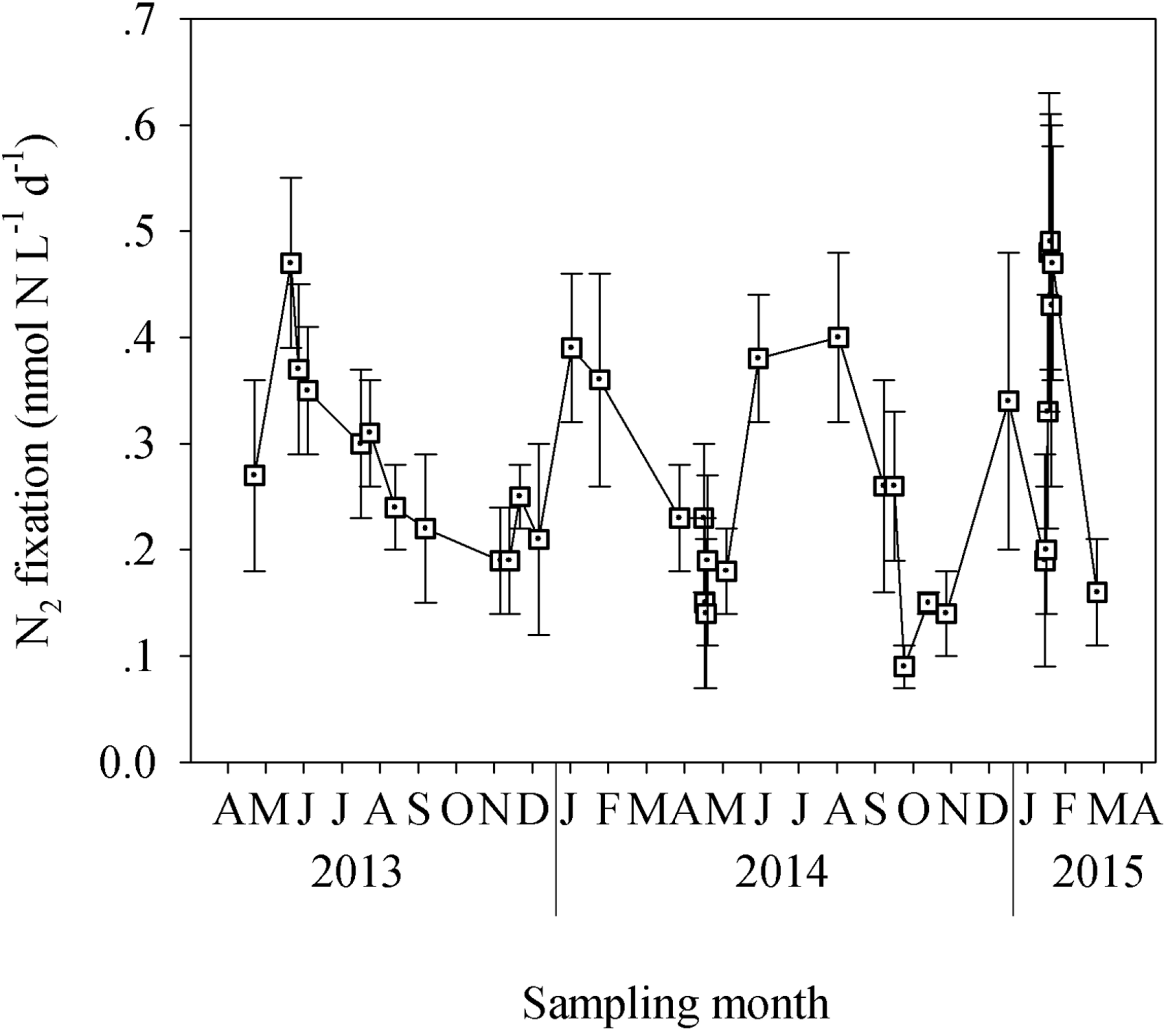
The temporal dynamics of N_2_ fixation in the coastal eastern Mediterranean Sea. Data was collected from April 2013 to April 2015, following 24 h incubations under ambient light and temperature.

Dinitrogen (N_2_) fixation rates exhibited a seasonal variability similar to that measured for heterotrophic bacterial abundance and bacterial production (Fig 4). The lowest N_2_ fixation rates were measured during spring and autumn, ranging between 0.1 and 0.2 nmol N L^-1^ d^-1^ (Fig 4). In contrast, 2–4 fold higher N_2_ fixation rates were recorded during the winter and early summer (~0.4 nmol N L^-1^ d^-1^, Fig 4), concurrent with the temporal dynamics of bacterial abundance and bacterial production temporal dynamics (Fig 3). New production due to N_2_ fixation equaled 10± 7% of the total primary production, with a larger contribution during wintertime (>8%) and the smallest contribution during autumn (<3%).

### The relationship between temperature and autotrophic and heterotrophic bacteria

**Fig 5:**
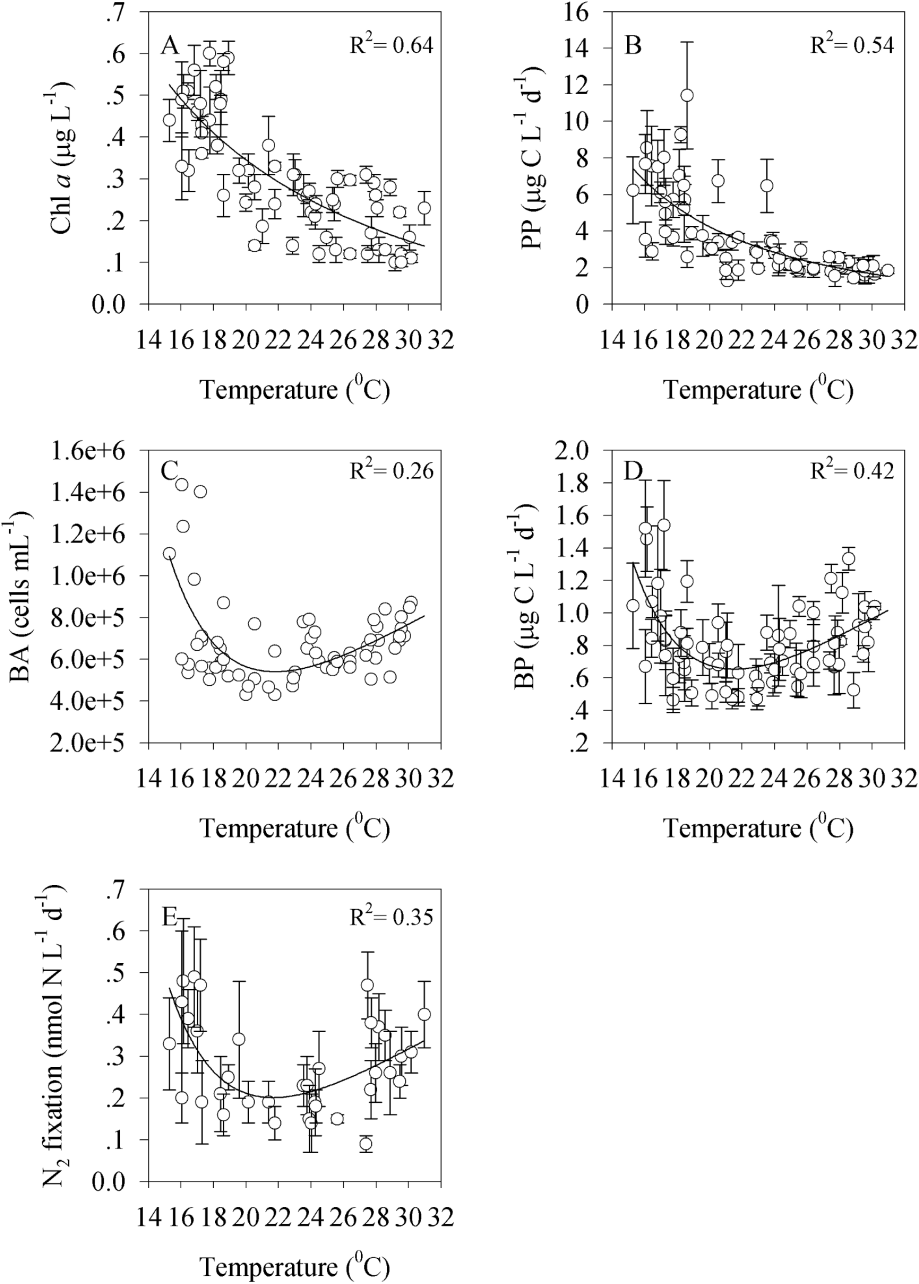
The relationship between temperature and bacterioplankton in the coastal eastern Mediterranean Sea. Data presented are for total Chl a (A), primary production (B), bacterial abundance (C), bacterial production (D) and N_2_ fixations (E) during the April 2013 to April 2015 sampling period.

The temporal distributions of Chl a and primary production correlated negatively (Pearson correlation, p< 0.001 and p< 0.0001 respectively) with temperature, and thus the largest algal biomass and the highest algal production were recorded during winter when the water temperature were the coldest (Fig 5A and 5B). In contrast, bacterial abundance, bacterial production and N_2_ fixation exhibited a different temporal pattern, with minimal abundances/rates during spring and autumn, when the surface water temperature was ~22°C, and maximal values during winter and summer (Fig 5C, 5D and 5E).

### The coastal versus the open Levantine surface waters

**Fig 6:**
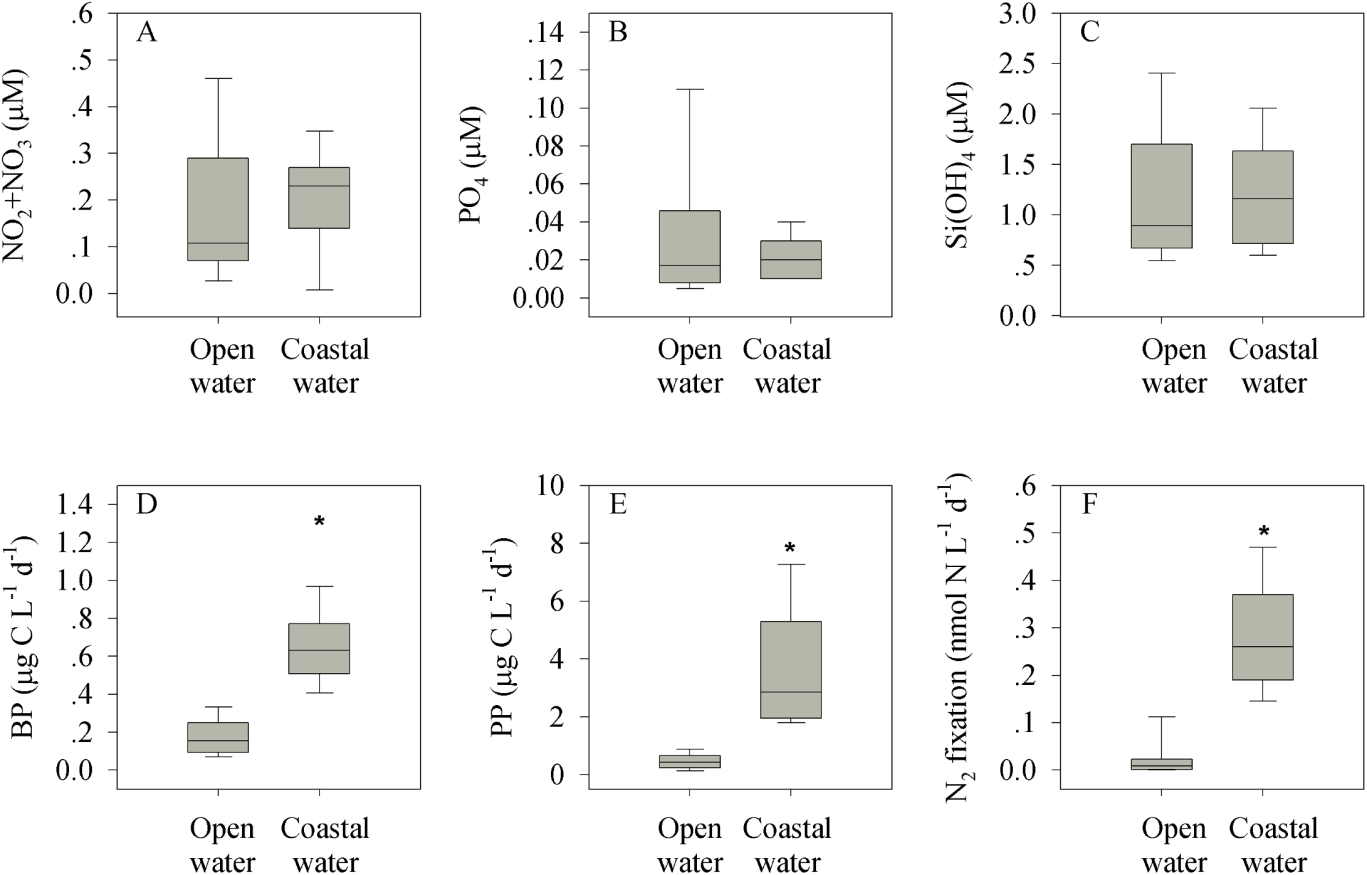
Comparison between the open and coastal Levantine Basin (eastern Mediterranean Sea) water. Box-plot distribution of NO_2_+NO_3_ (A), PO_4_ (B), Si(OH)_4_ (C), bacterial production, BP (D), primary production, PP (E) and N_2_ fixation (F) in the open Levantine Basin (euphotic zone, bottom depth of stations >1000 m) and in the coastal site (this study). Data for the open sea were compiled from Yogev et al., [35]; Kress et al., [3, 36], Rahav et al., [37, 38, 39]; Bonnet et al., [40], Ibello et al., [41] and Rahav et al., unpublished.

**Fig 7:**
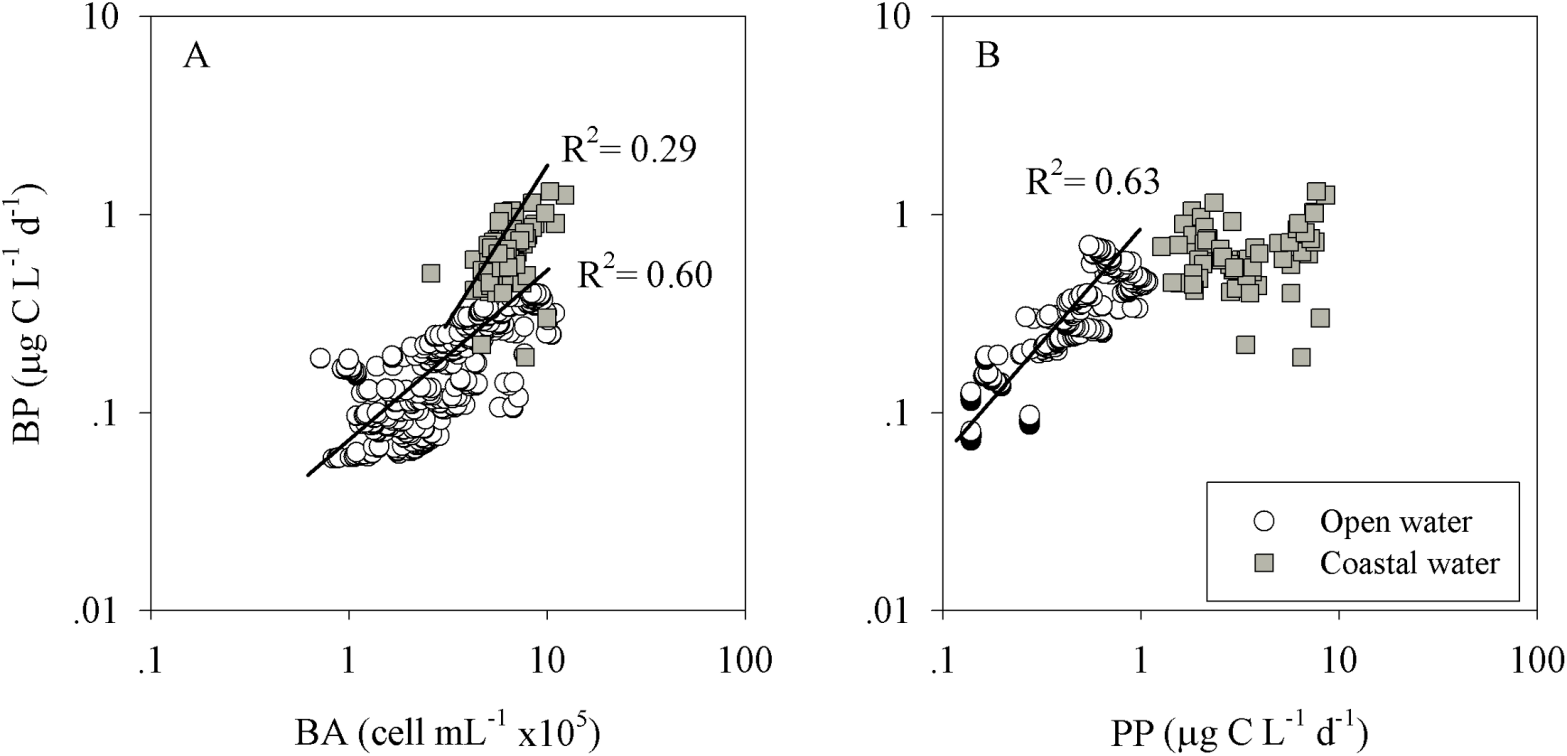
A log-log (base 10) relationship between bacterial production and bacterial abundance or primary production in the open and coastal Levantine Basin water. Data for the open sea stations (euphotic zone, bottom depth of stations >1000 m) were compiled from Zohary & Robarts [42], Zohary et al. [43, 44], Christaki et al. [45, 46], Turley et al. [47], Van Wambeke et al. [17, 48, 49], Ignatiades et al. [50], Kress et al. [51], Tanaka et al. [52, 53], Pulido-Villena et al. [15], and Rahav et al. [38]. A 95% confidence interval (CI) of the regressions is presented in Table 3.

In order to better understand the spatial dynamics of inorganic nutrients, production and N_2_ fixation rates in the Levantine Basin, we compared the data available from different studies conducted in the open (non-coastal) eastern Mediterranean Sea [3, 35, 36, 37, 38, 39, 40, 41] to the results obtained in this study (coastal). While inorganic nutrient concentrations were similar in both the coastal and the open water environments (P> 0.05, Fig 6A, 6B and 6C), autotrophic and heterotrophic bacterial production, as well as N_2_ fixation were significantly (P< 0.001) higher by threefold to sevenfold in the coastal waters (Fig 6D, 6E and 6F). Furthermore, heterotrophic bacteria were present in the same order of magnitude in the coastal water as they were in the open Levantine (average 3.9x 10^5^ cells mL^-1^), yet their averaged production rates were ~fourfold higher in the coastal (0.66 μg C L^-1^ d^-1^) relative to the open sea (0.17 μg C L^-1^ d^-1^, Fig 7A, Table 3) [42– 53]. This means that the average bacterial cell-specific activity (i.e. the bacterial production per bacterial cell) was ~50 pg C cell^-1^ d^-1^ in the open sea, whereas it was approximately twofold, in the coastal study site (106 pg C cell^-1^ d^-1^). Finally, the relationship between bacterial and primary production was examined in both water types [42– 53]. This relationship is a proxy for the flux of phytoplankton-derived carbon that passes through the microbial heterotrophic food web. While primary and bacterial production exhibited positive linear correlation in the open water of the Mediterranean Sea (p< 0.001), no such coupling was found in the coastal water (p = 0.114, Table 3, Fig 7B).

**Table 3:** The relationship between bacterial production (BP) and bacterial abundance (BA) or primary production (PP) in the open and coastal water of the Levantine Basin. Data for the open sea stations (euphotic zone, bottom depth of stations >1000 m) were compiled from Zohary & Robarts [4 2], Zohary et al. [4 3, 4 4], Christaki et al. [4 5, 4 6], Turley et al. [4 7], Van Wambeke et al. [1 7, 4 8, 4 9], Ignatiades et al. [50], Kress et al. [5 1], Tanaka et al. [5 2, 5 3], Pulido-Villena et al. [1 5], and Rahav et al. [38]. A 95% confidence interval (CI) is presented for the slope and the intercept.

		n	R^2^	Slope	Intercept	P value
Open water	BP vs. BA	383	0.60	0.03± 0.00	0.07± 0.01	<0.001
	BP vs. PP	223	0.63	1.19± 0.06	0.10± 0.02	<0.001
Coastal water	BP vs. BA	65	0.29	0.07± 0.01	0.19± 0.10	<0.001
	BP vs. PP	66	0.03	1.80± 1.13	2.41± 0.79	0.114
All data	BP vs. BA	448	0.60	0.03± 0.00	0.06± 0.01	<0.001
	BP vs. PP	289	0.38	4.29± 0.32	0.49± 0.15	<0.001

## Discussion

### Seasonal variability in the abundance and production of phytoplankton and bacteria

The seasonal dynamics of phytoplankton and bacterial properties are important to the ecology of all aquatic environments, as they are at the bottom of the food web and therefore hold a key role in nutrient recycling within the photic layers [54, 55, 56]. Their quantitative and qualitative variability can be associated with temperature-dependent physical processes such as stratification or mixing [57, 58], and with external nutrient inputs from natural and anthropogenic sources such as land runoff [59] and atmospheric dust depositions [60, 61].

In the present study, we used high-frequency sampling for a duration of two years (April 2013 to April 2015) in order to determine the phytoplankton and bacterial properties of the surface coastal waters of Tel Shikmona (eastern Mediterranean Sea) and to estimate some of the forcing factors that control their seasonality. While nutrient levels were usually very low (Table 1), the temperature varied greatly between the sampling periods (Fig 1), a fact which seems to explain the seasonal dynamics observed (Figs 2–5). Accordingly, temperature may affect primary and bacterial production rates [62, 63], increase or reduce algal and bacterial biomass [64], change species composition [65, 66], alter the timing and magnitude of annual blooms [65, 67] and influence sedimentation rates [68]. However, it should be noted that other physical and ecological processes that co-vary with the temperature (and nutrients) dynamics may also explain the variability and patterns of the phytoplankton and bacteria observed in this study. For example, an uncoupling between loss processes such as grazing or viral lysis and phytoplankton growth may result in an increase in phytoplankton during wintertime [69]. Thus, the increase in the abundance of cyanobacteria during the winter of 2014 and 2015 (Fig 2B), for example, may be explained by a weak coupling between grazers and phytoplankton and not by temperature or nutrient concentrations alone. Thus grazing rates and viral lysis in this system should be studied in relation to bacterioplankton spatial dynamics.

Picophytoplankton generally dominated the Chl a biomass according to their relative biomass and activity during summer (i.e. their portion out of all autotrophic biomass and activity, Table 2). Thus, autotrophic cyanobacteria such as *Synechococcus* and *Prochlorococcus* were most important to algal biomass during the summer months (Fig 2B, S1 Table), and were also the main contributors to primary production (Table 2, S1 Table). Similarly, picophytoplankton are the dominating phytoplankton in the upper layers of the pelagic eastern Mediterranean Sea waters [7, 39, 50, 70], as well as in other oligotrophic and subtropical environments [71, 72]. As water temperature declined during wintertime, larger autotrophic organisms (i.e., nano-microphytoplankton) became the major contributors to the Chl a biomass and total primary production (Table 2, S1 Table), even though the nutrient levels recorded were relatively low and did not substantially differ from the concentrations observed during the summer (Table 1). This means that either the balance between phytoplankton growth and loss was changed [69, 73], or that large autotrophs outcompeted smaller phytoplankton for the low levels of nutrients available or that other limiting elements were introduced by water mixing or land runoff. Indeed, following the sewage effluent discharge that occurred during February 2015, as evident from the increased nitrate and phosphate levels (Table 1) and the lower salinity (Fig 1), an immediate increase in large-sized Chl a and primary production was recorded (Fig 2), highlighting large size organisms benefit from the anthropogenic input more than small-size phytoplankton. Similarly, diatoms represent 37–58% of the >5 μm phytoplankton fraction during winter in the Cretan Sea [74], and 88% of the phytoplankton fraction at the shelf stations in the Levantine Basin [75]. This type of size-fractionated dynamic as was observed in this case between summer and winter, is common in many oceans [76], and likely occurs because of the imbalance processes detailed above [69], or because different species have an optimal nutrient uptake under different temperatures [77, 78, 79]. For example, PO_4_ uptake kinetics performed by *Synechococcus* are faster compared to those performed by most bacteria and algae [80, 81], allowing them to outcompete other species under low phosphorus concentrations. Regardless of the autotrophic size-fraction, the absolute level of Chl a was low, even during winter and spring (Fig 2A), hinting at the existence of other constraints, such as organic and inorganic nutrient sources or top-down control interactions. Furthermore, while Chl a and cyanobacteria exhibited a winter bloom that started at the end of October 2013 and lasted until March 2014 during the first year, during the following winter these variables moderately increased only in December 2014, and they reached a maxima only following the sewage effluents in February 2015 (Fig 2A and 2B). During October 2013, the water temperature was ~22°C, whereas at the same time in October 2014 it was higher than 26°C (Fig 1). This again suggests that temperature is an important controlling factor not only for the overall biomass and algal size distribution (Fig 2A and 2B, Table 2), but also for the timing of the bloom [65, 67]. However, we cannot rule out the possibility that other factor/s may have been involved in the phytoplankton bloom lag, among them top-down pressure. More baseline data is required in order to better understand this possible interannual variability, as well as the seasonal patterns. Such temporal lag in phytoplankton bloom between the two winter periods can shift the abundance, growth and activity of other components of the food web (such as grazers) and also change the oxygen consumption in the water column [82].

Heterotrophic bacterial abundance and bacterial production displayed seasonal patterns different from those of autotrophs, with peaks in abundance and activity during summer and winter and minimal activity during spring and autumn (Figs 3 and 5). We speculate that this seasonal cycle can largely be explained as a physiological response to the ambient temperature that prevails during summer, and, to a lesser extent, to the somewhat higher concentration of inorganic nutrients during winter. Bacterial metabolic rates vary linearly and positively with temperature in many coastal waters, such as Chesapeake Bay [83], the equatorial Pacific Ocean [84] and the Northern Baltic Sea [85], and therefore it is not surprising that the highest heterotrophic production rates and abundance were recorded when the water temperature was maximal. During wintertime, other constraints must have positively affected the heterotrophic bacteria, possibly interactions with large-size phytoplankton alongside the somewhat higher nutrient levels recorded, especially following the sewage input (Fig 2C). However, it should be noted that even an input of nutrient/s might result in different responses of the microbial community. Thus, Shiah and Ducklow [83] showed that nutrient limitation had acted differently on bacterial abundance, production and growth rates in Chesapeake Bay, and that the limitation is dependent on the season. Similarly, Morán et al., [86] showed in a study performed in the southern Bay of Biscay (NE Atlantic) that the sources and substrates used by heterotrophic bacteria differed seasonally. Thus, it is possible that the temporal dynamics of heterotrophic bacteria may also be explained by factors other than temperature and resource supply. All of these issues, including studying whether different bacterial groups interact with other microbial autotrophs or heterotrophs in the oligotrophic eastern Mediterranean habitat and if so how, should be investigated. It is possible, for example, that bacteria (autotrophic and/or heterotrophic) in this region have unique metabolic strategies that help them utilize the few nutrients available using different interactions. It was recently shown that heterotrophic bacteria in the eastern Mediterranean waters thrive on microenvironments such as transparent exopolymer particles, and it was even hypothesized that diazotrophs are likely to benefit from these microenvironment due to their low oxygen levels and high carbon content [39].

Dinitrogen fixation showed the same seasonal pattern that was observed for bacterial production or bacterial abundance (Fig 4). This type of relationship between these variables implies that heterotrophic bacteria may be an important component-fixing dinitrogen [37, 39]. Unlike the open eastern Mediterranean Sea, where the highest N_2_ fixation rates were measured during spring (~0.10 nmol N L^-1^ d^-1^) [37], coastal diazotrophy was the lowest in spring (Fig 4). This suggests that coastal diazotrophs and pelagic N_2_ fixers do not necessarily belong to the same groups, and/or that they have different metabolic controls and limitations, hence the temporal differences in their activities. Furthermore, similar to our measured coastal N_2_ fixation, which was the lowest during the autumn and the highest during summer, Foster et al., [87] reported undetectable rates during the fall and maximal rates during midsummer in the oligotrophic northern Red Sea. However, to date, our study is the first that shows full seasonal N_2_ fixation patterns in the eastern Mediterranean Sea (in both the open and the coastal habitats), and additional research is needed in this region to better understand the role of diazotrophs in providing new nitrogen in the coastal realm as well as studying interannual variability in N_2_ fixation.

### Autotrophic and heterotrophic bacterial communities; pelagic vs. coastal eastern Mediterranean Sea waters

The phosphorus and silicic acid levels measured in our coastal study site were similar to those routinely found in the surface of the pelagic eastern Mediterranean Sea, whereas nitrate was found to have a 2-fold higher mean in the coastal areas compared to our study site (Fig 6A, 6B and 6C). These overall low coastal nutrient concentrations reflect the oligotrophic nature of the eastern Mediterranean Sea, and yet highlight the fact that microorganisms (autotrophic or heterotrophic) inhabit the coastal water are exposed to higher nitrate concentrations, possibly via N_2_ fixation (Fig 4) or other outsource inputs such as ground water discharge or anthropogenic effluents. Contrary to the nutrient levels, primary production, bacterial production and N_2_ fixation were all significantly (P< 0.01) higher in the coastal water compared to their concentrations in the open sea (Fig 6D, 6E and 6F and references therein). Furthermore, the contribution of N_2_ fixation to new primary production, calculated based on the average particulate carbon to nitrogen ratio [35, 38], was ~twofold higher in the coastal stations than in the open sea. New production due to N_2_ fixation was 10± 7% of the total primary production at the coastal station, whereas it usually ranges between 0.5 and 2% in the open eastern Mediterranean Sea [35]. The higher contribution of N_2_ fixation to primary production in our study site is in agreement with measurements taken in more productive environments, such as the surface of the western Mediterranean Sea during spring (4–8%) [37], yet it was lower than the contribution reported in the core of an anticyclonic eddy (up to 35%) [40]. Our observations of the contribution of N_2_ fixation to primary production in the coastal eastern Mediterranean Sea suggest that diazotrophy may be an important source of bioavailable nitrogen that may support, at least to some extent, the metabolic needs of the microbial community. This is in contrast to the open eastern Mediterranean Sea, where N_2_ fixation is considered a negligible process [8, 35]. However, additional research is required to better understand the seasonal and interannual dynamics of diazotrophy and its relation with the microbial community in the eastern Mediterranean Sea.

The higher primary production, bacterial production and N_2_ fixation rates found in the coastal waters (Fig 6D, 6E and 6F), unlike the insignificant differences in the inorganic nutrients (Fig 6A, 6B and 6C) or phytoplankton abundance [5], can also be explained by factors other than elevated nitrate levels, including a different supply of other limiting nutrients that were not measured here (organic nutrients such as dissolved or particulate organic carbon, nitrogen, phosphorus/ phosphonates), top-down interactions, and the temperature. Whatever the controlling factors are, it seems that the microbial communities that inhabit the coastal areas have different metabolic rates and limitations. This is well exemplified in the bacterial abundance and bacterial production correlations plotted in both of these systems (Fig 7A). While the bacterial abundances measured in the present study comply with those measured in other studies of the eastern Mediterranean Sea (Fig 7A, Table 3 and references therein), the bacterial production rates were usually higher compared to those measured in open seawater (Fig 6 and references therein). Thus the heterotrophic cell-specific carbon uptake (bacterial production per cell) was higher in the coastal realm compared to the uptake measured in the open eastern Mediterranean Sea water (Fig 7). This indicates that coastal bacterial populations channel dissolved organic carbon into biomass twice as fast as bacteria do in the open sea, or that they have faster growth rates, indicating that coastal heterotrophs are more active and may have different metabolic requirements. Indeed, relatively high dissolved organic substrate concentrations are occasionally found in our coastal site [88] which can greatly contribute to bacterial proliferation in the eastern Mediterranean Sea [88, 89].

In order to further examine how nutrients affect the heterotrophic bacterial production rates, we inspected the relationship between bacterial production and bacterial abundance (Table 3, Fig 7). Assuming that bacterial production is a proxy for resource availability and that bacterial abundance is a proxy for bacterial biomass, the relationship between these two factors can indicate whether heterotrophic bacteria are a nutrient resource (bottom-up control) or whether they are controlled by predation (top-down) [18]. Based on the strong positive relationship (Table 3), we suggest that bacteria represent the prominent nutrient resource (bottom-up control) that controls the dynamics of abundance and production in this system. This trend was observed in both the coastal and the open eastern Mediterranean Sea waters (Table 3, Fig 7A and references therein), although the slope was shallower in open seawater sites (Table 3), indicating that the bacteria in these areas have slower carbon fixation rates per bacterial cell. However, to better characterize bacterial efficiency, bacterial growth rate measurements should be undertaken. Further, the results of a mesocosm (1-m^3^ bags) experiment conducted in May 2014 showed an insignificant difference (P> 0.05) in autotrophic (Chl a, primary production) and heterotrophic (bacterial abundance and production) variables between ambient and pre-filtered (63 μm) waters during 48 h incubations (S1 Fig). These findings suggest that micro-zooplankton had only a minor role in controlling phytoplankton and bacterial abundance/biomass and production during early summer, and indicate that the proliferation of these microorganisms is controlled by either nutrients (bottom-up control), physical properties (i.e. temperature), viral lysis or that they are grazed by small-size heterotrophic nano-flagellates. Moreover, when considering the constant presence of picophytoplankton in our study site throughout the year, and particularly during the summer months (Table 2), we are not surprised that micro-zooplankton are likely only minor contributors to grazing pressure and to limiting phytoplankton proliferation. The nutritional value of picophytoplankton is low compared to the larger phytoplankton (e.g., diatoms and dinoflagellates) that dominate the more productive habitats [46], and therefore fewer micro-zooplankton are expected to flourish, and the phytoplankton community is thus controlled more by nutrients (bottom-up) than by grazers (top-down).

### The relationship between bacterial production and primary production

The relationship between bacterial production and primary production is often used as an indicator of the flux of phytoplankton-derived carbon through the microbial heterotrophic food web [89, 90]. In principle, a positive relationship between bacterial production and primary production suggests that the latter is an important source for dissolved organic carbon that nourishes bacteria [15]. For example, the published data from the open Levantine Basin, compiled here, indicated a positive correlation between these variables (Table 3, Fig 7B). However, this relationship was not detected in the coastal waters examined in this study (Table 3), suggesting that coastal bacterial populations depend less on phytoplankton primary production for dissolved organic carbon. This suggest that heterotrophic microbial recycling processes likely predominate the coastal waters and/or that coastal heterotrophic bacteria have other sources of bacterial nutrition, possibly atmospheric inputs [91], or other anthropogenic pollutants (such as sewage) they can use to maintain their metabolism. Alternatively, this uncoupling between primary production and bacterial production may suggest a more complicated pathway by which organic carbon made by phytoplankton is processed via the food web and released into the dissolved pool where it is available to bacteria. Whatever the mechanism is, it is important to note that our study encompass all seasons, whereas the data for open sea was acquired mainly during the summer period. Therefore, in order to conduct a more seasonally unbiased comparison, further studies of the open sea-coastal water interactions should be conducted, focusing on seasonal variations in the eastern Mediterranean Sea with respect to vertical as well as integrated production. Moreover, more interannual studies should be carried out in order to distinguish between seasonal changes and long-term yearly variations.

## Conclusions

We show that the annual autotrophic and heterotrophic microbial abundances and production in the coastal eastern Mediterranean Sea were largely impacted by temperature and were less affected by the basal inorganic nutrient concentrations. These temperature-dependent trends might be especially important when considering the previously documented warming in the region [21] and the frequent extreme weather events observed in the eastern Mediterranean Sea in the last three decades [92]. Assuming water temperatures will continue to rise, and that summers will become longer, picophytoplankton will likely predominate throughout the year. Under these circumstances, seasonal variations will diminish. Moreover, picophytoplankton transfer very little energy to high trophic levels [46], and therefore the zooplankton that feed on them are likely to prevail, whereas large-size zooplankton’s abundance will most likely become scarce. These community shifts could have tremendous impacts on carbon sequestration in the eastern Mediterranean coast and are thus of great ecological interest. However, it is important to note that more studies are required in order to fully understand seasonal, and especially interannual, variability in bacterioplankton in the coastal easternmost Mediterranean Sea waters.

Finally, intrusion of coastal waters into the open sea, which is occasionally detected in the Levantine Basin through satellite observations [93], hyperspectral remote sensing surveys [60], or direct measurements [94], may export coastal bacteria and phytoplankton to the open sea, including potentially toxic cyanobacteria, diatoms, and dinoflagellates. Therefore, investigating the composition, abundance, and production of phytoplankton and bacteria in the coastal-open ocean interphase is essential if one is to understand the trophic balance in the eastern Mediterranean Sea.

## Supporting Information

S1 Fig Bottom-up and top-down interactions of autotrophic and heterotrophic microbial abundance and activity in the coastal eastern Mediterranean Sea- a mesocosm experiment. (DOCX)

S1 Table A summary table of all the data used to generate the results presented in this study. (DOCX)

## References

[1] Yacobi YZ, Zohary T, Kress N, Hecht A, Robarts RD, Waiser M, et al (1995) Chlorophyll distribution throughout the southeastern Mediterranean in relation to the physical structure of the water mass. J Mar Syst 6:179–190.

[2] Herut B, Almogi-Labin A, Jannink N, Gertman I (2000) The seasonal dynamics of nutrient and chlorophyll *a* concentrations on the SE Mediterranean shelf-slope. Oceanol Acta 23:771–782.

[3] Kress N, Gertman I, Herut B (2014) Temporal evolution of physical and chemical characteristics of the water column in the easternmost Levantine basin (eastern Mediterranean Sea) from 2002 to 2010. J Marine Syst 135:6–13.

[4] Krom MD, Emeis K-C, Van Cappellen P (2010) Why is the eastern Mediterranean phosphorus limited? Prog Oceanogr 85:236–244.

[5] Siokou-Frangou I, Christaki U, Mazzocchi MG, Montresor M, Ribera d’Alcalá M, Vaqué D, et al (2010) Plankton in the open Mediterranean Sea: a review. Biogeosciences 7:1543–1586.

[6] Bosc E, Bricaud A, Antoine D (2004). Seasonal and interannual variability in algal biomass and primary production in the Mediterranean Sea, as derived from 4 years of SeaWiFS observations. Global Biogeochem Cy 18, GB1005.

[7] Ignatiades L, Gotsis-Skretas O, Pagou K, Krasakopoulou E (2009) Diversification of phytoplankton community structure and related parameters along a large-scale longitudinal east-west transect of the Mediterranean Sea. J Plankton Res 31:411–428.

[8] Berman-Frank I, Rahav E (2012) Dinitrogen fixation as a source for new production in the Mediterranean Sea: a review In: Stambler N (ed) Life in the Mediterranean Sea: a look at habitat changes. Nova Science Publishers, New York, p 199–226.

[9] Moutin T, Raimbault P (2002) Primary production, carbon export and nutrients availability in western and eastern Mediterranean Sea in early summer 1996 (MINOS cruise). J Mar Syst 33–34:273–288.

[10] Marty J-C, Chiavérini J, Pizay M-D, Avril B (2002) Seasonal and interannual dynamics of nutrients and phytoplankton pigments in the western Mediterranean Sea at the DYFAMED time-series station (1991–1999). Deep Sea Res Pt II 49:1965–1985.

[11] Litchman E, Klausmeier CA, Schofield OM, Falkowski PG (2007) The role of functional traits and trade-offs in structuring phytoplankton communities: scaling from cellular to ecosystem level. Ecol Lett 10: 1170−1181. 17927770 10.1111/j.1461-0248.2007.01117.x

[12] Edwards KF, Thomas MK, Klausmeier CA, Litchman E (2012) Allometric scaling and taxonomic variation in nutrient utilization traits and maximum growth rate of phytoplankton. Limnol Oceanogr 57: 554−566.

[13] Magazzu G, Decembrini F (1995) Primary production, biomass and abundance of phototrophic picoplankton in the Mediterranean Sea: a review. Aquat Microb Ecol 9:97–104.

[14] Berman T, Townsend D, Elsayed S, Trees C, Azov Y (1984) Optical transparency, chlorophyll and primary productivity in the eastern Mediterranean near the Israeli coast. Oceanol Acta 7:367–372.

[15] Pulido-Villena E, Ghiglione JF, Ortega-Retuerta E, Van Wambeke F, Zohary T (2012) Heterotrophic bacteria in the pelagic realm of the Mediterranean Sea In: Stambler N (ed) Life in the Mediterranean Sea: a look at habitat changes. Nova Science Publishers, New York, p 227–265.

[16] Luna GM, Bianchelli S, Decembrini F, De Domenico E, Danovaro R, Dell’Anno A (2012) The dark portion of the Mediterranean Sea is a bioreactor of organic matter cycling. Global Biogeochem Cy 26 GB2017.

[17] Van Wambeke F, Christaki U, Bianchi M, Psarra S, Tselepides A (2000) Heterotrophic bacterial production in the Cretan Sea (NE Mediterranean). Prog Oceanogr 46:205–216.

[18] Billen G, Servais P, Becquevort S (1990) Dynamics of bacterioplankton in oligotrophic and eutrophic aquatic environments: bottom-up or top-down control? Hydrobiologia 207:37–42.

[19] Ducklow H (1992) Bacterial production and biomass in the oceans In: Kirchman DL (ed) Microbial Ecology of the Oceans. Wiley & Sons, New-York, p 85–120.

[20] Azov Y (1986) Seasonal patterns of phytoplankton productivity and abundance in nearshore oligotrophic waters of the Levant Basin (Mediterranean). J Plankton Res 8:41–53.

[21] Gertman I, Goldman R, Ozer T, Zodiatis G (2013) Interannual changes in the thermohaline structure of the southeastern Mediterranean. Rapp. Comm. int. Mer Médit., 40, 211.

[22] Krom MD, Kress N, Brenner S (1991) Phosphorus limitation of primary productivity in the eastern Mediterranean Sea. Limnol Oceanogr 36:424–432.

[23] Kress N, Herut B (2001) Spatial and seasonal evolution of dissolved oxygen and nutrients in the southern Levantine Basin (eastern Mediterranean Sea): chemical characterization of the water masses and inferences on the N:P ratios. Deep Sea Res Pt I 48:2347–2372.

[24] Holm-Hansen O, Lorenzen CJ, Holmes RW, Strickland JDH (1965) Fluorometric determination of chlorophyll. ICES J Mar Sci 30:3–15.

[25] Chisholm SW (1992) Phytoplankton size In: Falkowski PG and Woodhead AD (eds) Primary productivity and biogeochemical cycles in the sea. Plenum Press, New York, p 213–237.

[26] Vaulot D, Marie D (1999) Diel variability of photosynthetic picoplankton in the equatorial Pacific. J Geophys Res-Oceans 104:3297–3310.

[27] Simon N, Barlow RG, Marie D, Partensky F, Vaulot D (1994) Characterization of oceanic photosynthetic picoeukaryotes by flow cytometry. J Phycol 30:922–935.

[28] Robertson BR, Button DK, Koch AL (1998) Determination of the biomasses of small bacteria at low concentrations in a mixture of species with forward light scatter measurements by flow cytometry. Appl Environ Microbiol 64:3900–3909. 9758817 10.1128/aem.64.10.3900-3909.1998 PMC106576

[29] Stambler N (2006) Light and picophytoplankton in the Gulf of Eilat (Aqaba). J Geophys Res-Oceans 111:C11009.

[30] Steeman-Nielsen E (1952) The use of radioactive carbon (^14^C) for measuring organic production in the sea. J Cons Int Explor Mer 18:117–140.

[31] Simon M, Alldredge AL, Azam F (1990) Bacterial carbon dynamics on marine snow. Mar Ecol Prog Ser 65:205–211.

[32] Smith DC, Azam F (1992) A simple, economical method for measuring bacterial protein synthesis rates in seawater using ^3^H-leucine. Mar Microb Food Webs 6:107–114.

[33] Simon M, Azam F (1989) Protein-content and protein-synthesis rates of planktonic marine-bacteria. Mar Ecol Prog Ser 51: 201–213.

[34] Mohr W, Großkopf T, Wallace DWR, LaRoche J (2010) Methodological underestimation of oceanic nitrogen fixation rates. PLoS One 5:1–7. 10.1371/journal.pone.0012583 PMC2933240 20838446

[35] Yogev T, Rahav E, Bar-Zeev E, Man-Aharonovich D, Stambler N, Kress N, et al (2011) Is dinitrogen fixation significant in the Levantine Basin, east Mediterranean Sea? Environ Microb 13:854–871. 10.1111/j.1462-2920.2010.02402.x 21244595

[36] Kress N, Herut B, Gertman I (2012) In: Stambler N (ed) Life in the Mediterranean Sea: a look at habitat changes. Nova Science Publishers, New York, p 157–174.

[37] Rahav E, Herut B, Levi A, Mulholland MR, Berman-Frank I (2013) Springtime contribution of dinitrogen fixation to primary production across the Mediterranean Sea. Ocean Sci 9:489–498.

[38] Rahav E, Herut B, Stambler N, Bar-Zeev E, Mulholland MR, Berman-Frank I (2013) Uncoupling between dinitrogen fixation and primary productivity in the eastern Mediterranean Sea. J Geophys Res Biogeosci 118:195–202.

[39] Rahav E, Bar-Zeev E, Ohayon S, Elifantz H, Belkin N, Herut B, et al (2013) Dinitrogen fixation in aphotic oxygenated marine environments. Front Microbiol 4:227 10.3389/fmicb.2013.00227 23986748 PMC3753716

[40] Bonnet S, Grosso O, Moutin T (2011) Planktonic dinitrogen fixation along a longitudinal gradient across the Mediterranean Sea during the stratified period (BOUM cruise). Biogeosciences 8:2257–2267.

[41] Ibello V, Cantoni C, Cozzi S, Civitarese G (2010) First basin-wide experimental results on N_2_ fixation in the open Mediterranean Sea. Geophys Res Lett 37:1–5.

[42] Zohary T, Robarts RD (1998) Experimental study of microbial P limitation in the eastern Mediterranean. Limnol Oceanogr 43:387–395.

[43] Zohary T, Brenner S, Krom MD, Angel DL, Kress N, Li WKW, et al (1998) Buildup of microbial biomass during deep winter mixing in a Mediterranean warm-core eddy. Mar Ecol Prog Ser 167:47–57.

[44] Zohary T, Herut B, Krom MD, Fauzi C, Mantoura R, Pitta P, et al (2005) P-limited bacteria but N and P co-limited phytoplankton in the eastern Mediterranean—a microcosm experiment. Deep Sea Res Pt II 52:3011–3023.

[45] Christaki U, Van Wambeke F, Dolan JR (1999) Nanoflagellates (mixotrophs, heterotrophs and autotrophs) in the oligotrophic eastern Mediterranean: standing stocks, bacterivory and relationships with bacterial production. Mar Ecol Prog Ser 181:297–307.

[46] Christaki U, Van Wambeke F, Lefevre D, Lagaria A, Prieur L, Pujo-Pay M, et al (2011) Microbial food webs and metabolic state across oligotrophic waters of the Mediterranean Sea during summer. Biogeosciences 8:1839–1852.

[47] Turley CM, Bianchi M, Christaki U, Conan P, Harris JRW, Psarra S, et al (2000) Relationship between primary producers and bacteria in an oligotrophic sea–the Mediterranean and biogeochemical implications. Mar Ecol Prog Ser 193:11–18.

[48] Van Wambeke F, Christaki U, Giannokourou A, Moutin T, Souvemerzoglou K (2002) Longitudinal and vertical trends of bacterial limitation by phosphorus and carbon in the Mediterranean Sea. Microb Ecol 43:119–133. 11984634 10.1007/s00248-001-0038-4

[49] Van Wambeke F, Catala P, Pujo-Pay M, Lebaron P (2011) Vertical and longitudinal gradients in HNA-LNA cell abundances and cytometric characteristics in the Mediterranean Sea. Biogeosciences 8: 1853–1863.

[50] Ignatiades L, Psarra S, Zervakis V, Pagou K, Souvermezoglou E, Assimakopoulou G, et al (2002) Phytoplankton size-based dynamics in the Aegean Sea (eastern Mediterranean). J Mar Syst 36:11–28.

[51] Kress N, Thingstad TF, Pitta P, Psarra S, Tanaka T, Zohary T, et al (2005) Effect of P and N addition to oligotrophic eastern Mediterranean waters influenced by near-shore waters: a microcosm experiment. Deep Sea Res Pt II 52:3054–3073.

[52] Tanaka T, Zohary T, Krom MD, Law CS, Pitta P, Psarra S, et al (2007) Microbial community structure and function in the Levantine Basin of the eastern Mediterranean. Deep Sea Res Pt I 54:1721–1743.

[53] Tanaka T, Thingstad TF, Christaki U, Colombet J, Cornet-Barthaux V, Courties C, et al (2011) Lack of P-limitation of phytoplankton and heterotrophic prokaryotes in surface waters of three anticyclonic eddies in the stratified Mediterranean Sea. Biogeosciences 8:525–538.

[54] Reynolds C, Dokulil M, Padisák J (2000) Understanding the assembly of phytoplankton in relation to the trophic spectrum: where are we now? Hydrobiologia 424:147–152.

[55] Arrigo KR (2005) Marine microorganisms and global nutrient cycles. Nature 437:349–355. 16163345 10.1038/nature04159

[56] Jiao N, Herndl GJ, Hansell DA, Benner R, Kattner G, Wilhelm SW, et al (2010) Microbial production of recalcitrant dissolved organic matter: long-term carbon storage in the global ocean. Nat Rev Microbiol 8:593–599. 10.1038/nrmicro2386 20601964

[57] Grover JP, Chrzanowski TH (2006) Seasonal dynamics of phytoplankton in two warm temperate reservoirs: association of taxonomic composition with temperature. J Plankton Res 28:1–17.

[58] Garneau M-È, Roy S, Lovejoy C, Gratton Y, Vincent WF (2008) Seasonal dynamics of bacterial biomass and production in a coastal arctic ecosystem: Franklin Bay, western Canadian Arctic. J Geophys Res 113:C07S91.

[59] Beman JM, Arrigo K, Matson P (2005) Agricultural runoff fuels large phytoplankton blooms in vulnerable areas of the ocean. Nature 434:211–214. 15758999 10.1038/nature03370

[60] Herut B, Kress N, Tibor G (2002) The use of hyper-spectral remote sensing in compliance monitoring of water quality (phytoplankton and suspended particles) at ‘hot spot’ areas (Mediterranean coast of Israel). Fresen Environl Bull 11:782–787.

[61] Paytan A, Mackey RM, Chen Y, Lima ID, Doney SC, Mahowald N, et al (2009) Toxicity of atmospheric aerosols on marine phytoplankton. P Natl Acad Sci USA 106:4601–4605. 10.1073/pnas.0811486106 PMC2653564 19273845

[62] Adams HE, Crump BC, Kling GW (2010) Temperature controls on aquatic bacterial production and community dynamics in arctic lakes and streams. Environ Microbiol 12:1319–1333. 10.1111/j.1462-2920.2010.02176.x 20192972

[63] Tait LW, Schiel DR (2013) Impacts of temperature on primary productivity and respiration in naturally structured macroalgal assemblages. PLoS One 8:e74413 10.1371/journal.pone.0074413 24058560 PMC3772813

[64] Li WKW, Dickie PM, Spry JA (1998) Plankton monitoring program in the Bedford Basin, 1991–1997. Can Data Rep Fish Aquat Sci 0:1–342.

[65] Hoshiai G-i, Suzuki T, Kamiyama T, Yamasaki M, Ichimi K (2003) Water temperature and salinity during the occurrence of *Dinophysis fortii* and *D*. *acuminata* in Kesennuma Bay, northern Japan. Fish Sci 69:1303–1305.

[66] Li WKW, McLaughlin FA, Lovejoy C, Carmack EC (2009) Smallest algae thrive as the Arctic Ocean freshens. Science 326:539 10.1126/science.1179798 19900890

[67] Harris GP, Griffiths FB, Clementson LA, Lyne V, Van der Doe H (1991) Seasonal and interannual variability in physical processes, nutrient cycling and the structure of the food chain in Tasmanian shelf waters. J Plankton Res 13:109–131.

[68] Cederwall H, Elmgren R (1990) Biological effects of eutrophication in the Baltic Sea, particularly the coastal zone. AMBIO 19:109–112.

[69] Behrenfeld MJ (2010) Abandoning Sverdrup's Critical Depth Hypothesis on phytoplankton blooms. Ecology 91:977–989. 20462113 10.1890/09-1207.1

[70] Denis M, Thyssen M, Martin V, Manca B, Vidussi F (2010) Ultraphytoplankton basin-scale distribution in the eastern Mediterranean Sea in winter: link to hydrodynamism and nutrients. Biogeosciences 7:2227–2244.

[71] Takahashi M, Hori T (1984) Abundance of picophytoplankton in the subsurface chlorophyll maximum layer in subtropical and tropical waters. Mar Biol 79:177–186.

[72] Li WKW (2002) Macroecological patterns of phytoplankton in the northwestern North Atlantic Ocean. Nature 419:154–157. 12226662 10.1038/nature00994

[73] Chen B, Liu H (2010) Trophic linkages between grazers and ultraplankton within the microbial food web in subtropical coastal waters. Mar Ecol Prog Ser 407: 43–53.

[74] Gotsis-Skretas O, Pagou K, Moraitou-Apostolopoulou M, Ignatiades L (1999) Seasonal horizontal and vertical variability in primary production and standing stocks of phytoplankton and zooplankton in the Cretan Sea and the Straits of the Cretan Arc (March 1994-January 1995). Prog Oceanogr 44:625–649.

[75] Psarra S, Tselepides A, Ignatiades L (2000) Primary productivity in the oligotrophic Cretan Sea (NE Mediterranean): seasonal and interannual variability. Prog Oceanogr 46:187–204.

[76] Kostadinov TS, Siegel DA, Maritorena S (2010) Global variability of phytoplankton functional types from space: assessment via the particle size distribution. Biogeosciences 7: 3239–3257.

[77] Townsend DW, Cammen LM, Holligan PM, Campbell DE, Pettigrew NR (1994) Causes and consequences of variability in the timing of spring phytoplankton blooms. Deep Sea Res Pt I 41:747–765.

[78] Oviatt C, Keller A, Reed L (2002) Annual primary production in Narragansett Bay with no bay-wide winter-spring phytoplankton bloom. Estuar Coast Shelf Sci 54:1013–1026.

[79] Behrenfeld MJ (2010) Abandoning Sverdrup’s critical depth hypothesis on phytoplankton blooms. Ecology 91:977–989. 20462113 10.1890/09-1207.1

[80] Moutin T, Thingstad T, Van Wambeke F, Marie D, Slawyk G, Raimbault P, et al (2002) Does competition for nanomolar phosphate supply explain the predominance of the cyanobacterium *Synechococcus*? Limnol Oceanogr 47:1562–1567.

[81] Tanaka T, Rassoulzadegan F, Thingstad T (2003) Measurements of phosphate affinity constants and phosphorus release rates from the microbial food web in Villefranche Bay, northwestern Mediterranean Limnol Oceanogr 48:1150–1160.

[82] Lomas MW, Bates NR, Johnson RJ, Knap AH, Steinberg DK, Carlson CA (2013) Two decades and counting: 24-years of sustained open ocean biogeochemical measurements in the Sargasso Sea. Deep Sea Res Pt II 93: 16–32

[83] Shiah F-K, Ducklow HW (1994) Temperature regulation of heterotrophic bacterioplankton abundance, production, and specific growth rate in Chesapeake Bay. Limnol Oceanogr 39:1243–1258.

[84] Kirchman DL, Rich JH (1997) Regulation of bacterial growth rates by dissolved organic carbon and temperature in the equatorial Pacific Ocean. Microb Ecol 33:11–20. 9039761 10.1007/s002489900003

[85] Pinhassi J, Hagström Å (2000) Seasonal succession in marine bacterioplankton. Aquat Microb Ecol 21:245–256.

[86] Morán XAG, Calvo-Diaz A, Ducklow HW (2010) Total and phytoplankton mediated bottom-up control of bacterioplankton change with temperature in NE Atlantic shelf water. Aquat Microb Ecol 58: 229–239.

[87] Foster RA, Paytan A, Zehr JP (2009) Seasonality of N_2_ fixation and nifH gene diversity in the Gulf of Aqaba (Red Sea). Limnol Oceanogr 54: 219–233.

[88] Bar-Zeev E, Rahav E (2015) Microbial metabolism of transparent exopolymer particles during the summer months along a eutrophic estuary system. Front Microbiol 6:403 10.3389/fmicb.2015.00403 26042092 PMC4436900

[89] Bar-Zeev E, Berman T, Rahav E, Dishon G, Herut B, Kress N, et al (2011) Transparent exopolymer particle (TEP) dynamics in the eastern Mediterranean Sea. Mar Ecol Prog Ser. 431:107–118.

[90] Cole JJ, Findlay S, Pace ML (1988) Bacterial production in fresh and saltwater ecosystems: a cross-system overview. Mar Ecol Prog Ser 43:1–10.

[91] Herut B, Zohary T, Krom MD, Mantoura FR, Pitta P, Psarra S, et al (2005) Response of East Mediterranean surface water to Saharan dust: On-board microcosm experiment and field observations. Deep Sea Res Pt II 52: 3024–3040.

[92] Füssel H-M (2009) An updated assessment of the risks from climate change based on research published since the IPCC Fourth Assessment Report. Climatic Change 97:469–482.

[93] Groom S, Herut B, Brenner S, Zodiatis G, Psarra S, Kress N, et al (2005) Satellite-derived spatial and temporal biological variability in the Cyprus Eddy. Deep Sea Res Pt II 52:2990–3010.

[94] Efrati S, Lehahn Y, Rahav E, Kress N, Herut B, Gertman I, et al (2013) Intrusion of coastal waters into the pelagic eastern Mediterranean: *in situ* and satellite-based characterization. Biogeosciences 10:3349–3357.

