# Supplementary material for: The Temporal Dynamics of Coastal Phytoplankton and Bacterioplankton in the Eastern Mediterranean Sea
Source: PLoS One. 2015 Oct 16;10(10):e0140690. doi: 10.1371/journal.pone.0140690 (PMC4608699; doi:10.1371/journal.pone.0140690)
Supplement: S1 Fig — (DOCX) [file pone.0140690.s001.docx]

**Mesocosm preparation and setup**

Acid-washed polyethylene bags (6 bags, each 1 m^3^), supported by cylindrical plastic frames, were deployed within a continuously circulating seawater 16 m^3^ pool in order to maintain the ambient temperature of the water during May 2014. Surface (2 m deep) coastal seawater from Tel Shikmona (from the same location as the on-going samplings described in the main text) was pumped and distributed homogenously between the mesocosm bags. Filling the bags required 2 h. Polyethylene covers prevented seawater from evaporating or from entering the bags, and also prevented external contamination, such as atmospheric dust. The mesocosms were located at the National Institute of Oceanography of the Israel Oceanographic and Limnological Research (IOLR) in Haifa, Israel.

**Experimental design**

A mesocosm experiment was conducted for a total of 48 h, starting on May 24^th^ 2014, in order to represent early summer characteristics. One of the goals of this mesocosm experiment was to characterize the role of grazers in natural autotrophic and heterotrophic abundance and in activities that dominated the coastal waters at the time of the study. Thus, we filled triplicate mesocosm bags with pre-filtered seawater (50 µm) in order to remove most of the zooplankton fraction and compared it to triplicate mesocosms bags filled with ambient waters that were not pre-filtered. We qualitatively estimated the zooplankton abundance in the mesocosms using a stereomicroscope (Nikon SMZ 745T) to ensure that the zooplankton content of the pre-filtered bags was sufficient.

The initial characteristics of the seawater used to fill the bags, including chlorophyll (Chl.*a*), primary productivity (PP), bacterial abundance (BA), and bacterial productivity (BP), were measured (i.e., time zero) as described in detail in the main text. Subsequent samples were acquired 6 h, 24 h, and 48 h later for the same variables mentioned above.

**Results**

The initial water properties during May 2014 exhibited early summertime characteristics. The Chl.*a* concentration was relatively low, 0.26 ± 0.17 µg L^-1^, and was followed by low primary productivity, 3.46 ± 0.32 µg C L^-1^ d^-1^ (Fig. S1). Heterotrophic bacterial biomass averaged 6.98×10^5^ ± 0.54×10^5^ cells mL^-1^ and bacterial productivity was 0.69 ± 0.59 µg C L^-1^ d^-1^ (Fig. S1). Based on the stereomicroscopic estimations, ~80% of copepods, isopods, and other zooplankton species were removed by the 50-µm net.

The removal/reduction of zooplankton (filtered mesocosms) did not stimulate significant changes in the autotrophic and heterotrophic variables (p > 0.05), except for Chl.*a*, since after 6 h of incubation, significantly lower concentrations were measured in the “filtered” bags, compared to the initial (p = 0.04) and unfiltered 6 h bags (p = 0.01). Although not significant, in most cases Chl.*a* and bacterial abundance were higher in the “filtered” mesocosms compared to the untreated controls, suggesting weak grazing pressure. Concurrently, both primary and bacterial productivity were usually higher in the “low- grazers” mesocosms. It is thus possible that longer incubation periods are required to fully estimate the actual grazing pressure on phytoplankton and bacteria. Yet, within these short time scales (two days max), grazing was not a significant factor for both bacteria and phytoplankton. Our results suggest that low grazing pressure (i.e. “top-down control”) occurred during the timeframe of our study, at least during summer, although additional research, performed during different seasons (particularly winter), is required. Furthermore, longer incubation periods, improved size-fractionated filtration (i.e. different cutoffs) and more repetitions are required to better understand food web dynamics and controls.





**S1 Fig.** The dynamics of Chl.*a* (A), primary productivity (B), bacterial abundance (C), and bacterial productivity (D) in unfiltered mesocosms (controls, white bars) versus pre-filtered seawater (> 63 µm, stripe bars) after 6 h, 24 h, and 48 h incubations, performed during May 2014 in Tel-Shikmona.
