# Supplementary material for: The Temporal Dynamics of Coastal Phytoplankton and Bacterioplankton in the Eastern Mediterranean Sea
Source: PLoS One. 2015 Oct 16;10(10):e0140690. doi: 10.1371/journal.pone.0140690 (PMC4608699; doi:10.1371/journal.pone.0140690)
Supplement: S1 Table — (DOCX) [file pone.0140690.s002.docx]

**S1 Table.** Summary of the results presented in the main paper.
